# Double-balloon catheter for induction of labour in women with a previous cesarean section, could it be the best choice?

**DOI:** 10.1007/s00404-017-4343-7

**Published:** 2017-03-18

**Authors:** Carlos De Bonrostro Torralba, Eva Lucía Tejero Cabrejas, Sabina Marti Gamboa, María Lapresta Moros, Jose Manuel Campillos Maza, Sergio Castán Mateo

**Affiliations:** 1 Hospital Universitario Materno-Infantil Miguel Servet, Paseo Isabel La Católica 1-3, 50009 Zaragoza, Spain; 2 Department of Obstetrics, Hospital Universitario Materno-Infantil Miguel Servet, Zaragoza, Spain

**Keywords:** Induced Labour, Vaginal birth after cesarean, Cervical ripening, Obstetric labour, Trial of labour, Catheters

## Abstract

**Introduction:**

We analysed the efficacy and safety of double-balloon catheter for cervical ripening in women with a previous cesarean section and which were the most important variables associated with an increased risk of repeated cesarean delivery.

**Materials and methods:**

We designed an observational retrospective study of 418 women with unfavourable cervices (Bishop Score <5), a prior cesarean delivery, and induction of labour with a double-balloon catheter. Baseline maternal data and perinatal outcomes were recorded for a descriptive, bivariate, and multivariate analysis. A *p* value <0.05 was considered statistically significant.

**Results:**

Most women improved their initial Bishop Score (89.5%) although only a 20.8% of them went into spontaneous active labour. Finally, 51.4% of the women achieved a vaginal delivery. Five cases of intrapartum uterine rupture (1.2%) occurred. After multivariate analysis, main risk factors for repeated cesarean section were dystocia in the previous pregnancy (OR 1.744; CI 95% 1.066–2.846), the absence of previous vaginal delivery (OR 2.590; CI 95% 1.066–6.290), suspected fetal macrosomia (OR 2.410; CI 95% 0.959–6.054), and duration of oxytocin induction period (OR 1.005; CI 95% 1.004–1.006). The area under the curve was 0.789 (*p* < 0.001).

**Conclusions:**

Double-balloon catheter seems to be safe and effective for cervical ripening in women with a previous cesarean delivery and unfavourable cervix. In our study, most women could have a vaginal delivery in spite of their risk factors for cesarean delivery. A multivariate model based on some clinical variables has moderate predictive value for intrapartum cesarean section.

## Introduction

Induction of labour is a common obstetric intervention that occurs in a high proportion of pregnancies [1]. This procedure tries to obtain a successful vaginal birth in women with an unfavourable cervix by modifying its characteristics [2]. There are several methods for cervical ripening that have been used over the years, but both mechanical and pharmacological devices seem to be effective for promoting the onset of labour [3].

Women with a prior cesarean section (PCS) are more and more frequent in our clinical practice and 17.6% of them require labour induction [4]. Unfortunately, a previous uterine scar seems to be linked to a higher risk of having a cesarean delivery and complications such as uterine rupture in subsequent pregnancies, events that are more common when there is no spontaneous onset of labour [5].

Induction of labour in women who had a PCS should be managed with caution according to the special complications that they can suffer. Uterine rupture is one of the most terrible consequences of trial of labour (TOL) and using prostaglandins for cervical ripening may have a sixfold increased risk [6]. Specifically, misoprostol is a pharmacological agent that is not recommended for induction of labour in this clinical context [7].

Mechanical methods of induction are becoming an option to improve outcomes in women with PCS. Several trials have shown that may be as effective in labour induction as pharmacological methods with lower rates of uterine hyperstimulation [8]. In women with a PCS, the use of mechanical methods may be associated with a lower rate of uterine rupture [9, 10]. Most of these studies used Foley catheter for induction of labour, but there are not many references using the double-balloon catheter (DBC) in women with a PCS [9, 11].

DBC is a mechanical device with indication for induction of labour in pregnant women. This catheter has two balloons (intrauterine and intravaginal balloons) that can be filled with a maximum volume of saline fluid of 80 cc for each one.

It is placed in cervical canal during 12–24 h in cases in which the DBC did not fall spontaneously. It is recommended to remove the device in case of ruptured amniotic membranes [12].

The primary objective of this study was to analyse safety and efficacy of the DBC for cervical ripening in women with PCS and which were the variables predicting an increased risk of cesarean delivery.

## Materials and methods

We designed an observational retrospective study including women with PCS requiring induction of labour. They were recruited in Miguel Servet Hospital in Zaragoza (Spain) between January 2009 and December 2015, both months included. Our medical centre is a tertiary hospital that conducts over 4000 deliveries each year. Cesarean delivery rates during this period have been between 14.7 and 16.5% of all deliveries. Our vaginal delivery rate for women with PCS has fluctuated between 56.3 and 59.5% during this recruitment period.

### Selection criteria

Pregnant women over 34^+ 0^ weeks of gestation with a single PCS (segmental transverse uterine scar) with singleton gestation in cephalic presentation, intact membranes, and Bishop score of 0–4 were eligible for inclusion. Exclusion criteria were maternal age <18 years, previous transmural uterine surgery (different from segmental transverse cesarean section), more than one PCS, premature ruptured amniotic membranes, abnormal fetal heart rate tracing, or any contraindication for vaginal birth.

### Clinical management

Women were given information about this mechanical method of preinduction, and risks and benefits about TOL were discussed. Informed consent was obtained on last prenatal visit and it was confirmed on admission for induction of labour.

A cervical digital examination was performed before the DBC was placed and Bishop score at that moment was recorded. If the score was lower than five points, a DBC was used for cervical ripening, filling both balloons up to 80 cc of saline. If pain was felt during the process, complete filling of the balloons was delayed for some minutes. Once the DBC was correctly placed, it was strapped to the inner aspect of one leg without traction.

The catheter was removed after 12 h if the initial Bishop Score was 3–4 points, and after 24 h if Bishop Score was 0–2 points, if spontaneous expulsion had not occurred.

If cervical score was higher, oxytocin induction was indicated. Double-balloon catheter was the only preinduction method used in these cases, since vaginal prostaglandins are not used in women with a prior uterine scar in our hospital.

Discontinuous fetal heart rate monitoring was performed during preinduction process. Indications for removal of the catheter were discomfort for the woman, ruptured amniotic membranes, onset of active labour, or non-reassuring fetal heart pattern. Oxytocin infusion with artificial rupture of amniotic membranes was used in case that active labour did not occur after that period of time.

Labour was managed by the attending obstetricians and midwives according to existing protocols in the hospital. Analgesia was administered at maternal request. Continuous fetal heart rate monitoring was used during oxytocin induction and active labour. Fetal scalp blood sampling was undertaken by the obstetric team if a non-reassuring fetal heart rate pattern appeared.

### Statistical methods

Study data were collected from delivery information that was recorded by the research team. Maternal and neonatal data were recorded and collected for posterior statistical analysis. IBM Statistics Process Social Sciences 22.0 for Mac (Copyright© SPSS Inc., 2013) was used.

Outcome data are presented as percentages, medians, and mean values. Comparison between groups was analysed using the Chi-square, Fischer’s exact, *t* Student, and Mann–Whitney tests. For multivariate analysis, Naegelkerke *r* ^2^ was calculated and Hormer or Lemeshow test performed. Multiple lineal regression was used by calculating *R* ^2^ and Durbin-Watson test. A *p* value < 0.05 was considered statistically significant.

### Ethics approval

This study has the approval of the local ethics committee (Act N. 15/2016, Comité Ético de Investigación Clínica de Aragón, CEICA).

## Results

### Recruitment and baseline data

Over the study period, 2235 women with previous cesarean section had a trial of labour in our hospital. Of them, 460 women (20.5%) initiated a mechanical induction of labour. However, 42 women (9.13%) were excluded because of not meeting selection criteria. There were no losses to follow-up and complete information required was available in all cases.

**Fig. 1:**
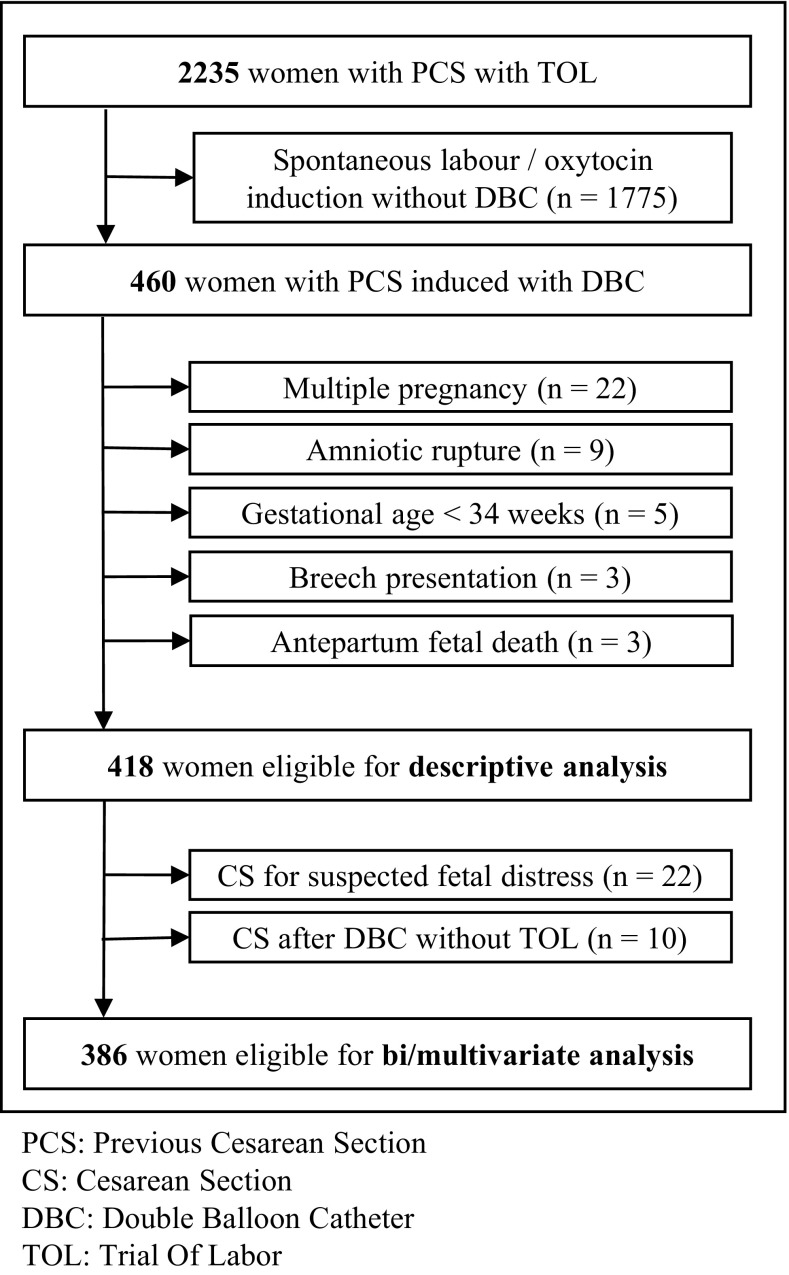
Recruitment and flow of women with cervical ripening with DBC

For multivariate analysis to predict repeated cesarean section because of intrapartum dystocia, we decided to exclude those women that did not initiate oxytocin induction of labour after the DBC removal (10 women, 2.4%) and those who had a cesarean section because of a suspected fetal distress (22 women, 5.3%). Finally, 386 women were included for multivariate analysis (Fig. 1).

Gestational age at the moment of initiating preinduction process was higher than 40^+ 0^ in 68.4% of the total sample. In fact, the main indication for induction of labour was prolonged pregnancy. Most of these women had not a previous vaginal delivery and the initial Bishop score was less than five points in all cases. Median value for the initial Bishop Score before using DBC was two points (Table 1).

**Table 1:** Baseline characteristics (*n* = 418)

Age^a^	34 (6)
History of previous vaginal delivery^b^	52 (12.4)
Dystocia^c^ as indication for PCS^b^	179 (45.3)
Gestational age ^b^	
<37^+ 0^ weeks	19 (4.5)
37^+ 0^–39^+ 6^	113 (27)
40^+ 0^–42^+ 0^	286 (68.4)
Indication for induction (%)^b^	
Postdates	254 (60.8)
Hypertensive disease	35 (8.4)
Fetal macrosomia and maternal diabetes	33 (7.9)
Gestational diabetes	32 (7.7)
Other maternal indication	25 (5)
Intrauterine growth restriction	15 (3.6)
Other fetal indication	13 (3.1)
Oligohydramnios	11 (2.6)
Initial Bishop Score^a^	2 (2)
Initial Bishop score (detailed)^b^	
0	21 (5)
1	143 (34.2)
2	126 (30.1)
3	88 (21.1)
4	40 (9.6)

*PCS* previous cesarean section

^a^Data expressed as median (interquartile range)

^b^Data expressed as *n* (%)

^c^Dystocia included failed induction, failure to progress, or suspected intrapartum cephalopelvic disproportion

### Preinduction and delivery outcomes

A majority of the women undergoing TOL with DBC had cervical changes in Bishop score and even 20.8% of them went into active labour during preinduction process. After the DBC was removed, 61% of the women had reached a Bishop score higher than four points. Median value for Bishop score after removing DBC was five.

In 44 women (10.5%), there were no cervical changes after the use of the mechanical device (Table 2). In nine of these women, cesarean section was indicated after the removal of DBC because of the absence of cervical changes and particularly unfavourable cervical characteristics (Bishop score lower than two points in all cases). Those remaining 35 women with no cervical modifications had higher Bishop scores and underwent oxytocin induction. Finally, 14 of them (40%) had a vaginal delivery. An urgent cesarean delivery was needed in one woman because of an abruptio placentae occurred during the preinduction process.

**Table 2:** Preinduction and delivery outcomes (*n* = 418)

Preinduction outcomes	
Bishop score before DBC^a^	2 (2)
Bishop score after DBC^a^	5 (3)
Bishop score after DBC (detailed)^b^	
0–4	163 (39.0)
>4	255 (61.0)
Change in Bishop score^a^	3 (2)
Active labour during DBC^b^	87 (20.8)
Absence of cervical changes^b^	44 (10.5)
Delivery outcomes	
Time from induction to delivery (hours)^a^	30.3 (8.5)
Intrapartum fever^b^	44 (10.5)
Fetal scalp blood sample^b^	72 (17.2)
Meconium-stained amniotic fluid^b^	57 (13.6)
Intrapartum non-reassuring FHR tracing^b^	90 (21.5)
Mode of delivery^b^	
Spontaneous vaginal delivery	117 (28.0)
Assisted vaginal delivery	98 (23.4)
Cesarean section	203 (48.6)
Indication for cesarean section^b^	
Failed induction	85 (41.9)
Failure to progress	75 (36.9)
Suspected intrapartum fetal distress	23 (11.3)
Suspected cephalopelvic disproportion	20 (9.9)
Indication for assisted vaginal delivery^b^	
Prolonged second stage of labour	62 (63.2)
Suspected intrapartum fetal distress	26 (26.5)
Fetal head rotation dystocia	10 (10.2)
Birthweight (grams)^a^	3455 (678)
Apgar Score <4 at 1 min^b^	8 (1.9)
Apgar Score <7 at 5 min^b^	4 (0.9)
Umbilical artery pH < 7.00	5 (1.2)

*FHR* fetal heart rate, *DBC* double-balloon catheter

^a^Data expressed as median (interquartile range)

^b^Data expressed as *n* (%)

Maternal fever was present in 10.5% of the women and a fetal scalp blood sample was required in 17.2% of them. Intrapartum non-reassuring fetal heart rate pattern was present in 21.5% of all deliveries. Finally, 48.6% of women had a cesarean section delivery, mostly due to a failed induction of labour or failure to progress. Cesarean section because of a suspected fetal distress occurred in 11.3% of all the women. An assisted vaginal delivery was required in 23.4% of the women; most of them due to a prolonged second stage of labour.

**Fig. 2:**
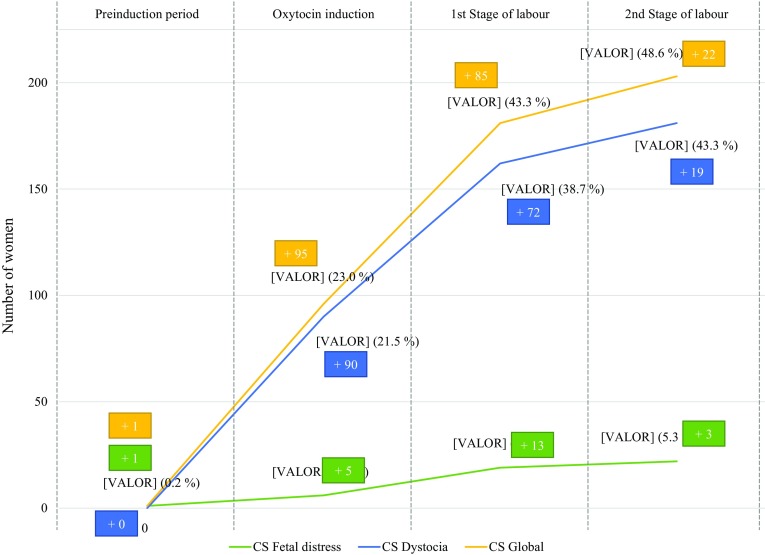
Cesarean delivery rate during preinduction, induction, and active labour

Median time from induction to delivery was 30.3 h. Apgar Score at 5 min was lower than seven in four neonates (Table 2). The risk of having an intrapartum cesarean section was different depending on the stage of labour. In fact, the probability of having a cesarean delivery was lower if the women had reached complete cervical dilation (Fig. 2).

Regarding maternal complications, intrapartum uterine rupture was diagnosed in five women, one of them required an obstetric hysterectomy, because the torn uterus could not be successfully repaired from a conservative approach. However, none of the uterine ruptures happened during the use of DBC, but rather during the active labour.

In four cases, amniotic membranes were ruptured, while the catheter was being used, so it had to be removed and begin an oxytocin induction. There were three cases of abruptio placentae, one of them occurring during preinduction process and leading to an urgent cesarean section. Removal of the DBC and oxytocin induction was required in two cases because of abnormalities in fetal heart rate tracing during the preinduction period. Main maternal adverse outcomes were infrequent (Table 3).

**Table 3:** Maternal complications (*n* = 418)

Uterine rupture	5 (1.2)
Ruptured amniotic membranes during DBC	4 (0.9)
Uterine atony	4 (0.9)
Abruptio placentae	3 (0.7)
Bladder tear	2 (0.5)
Non-reassuring FHR during DBC	2 (0.5)
Maternal blood transfusion	2 (0.5)
Perineal tear III–IV degree	2 (0.5)
Perineal hematoma	1 (0.2)
Postpartum bladder atony	1 (0.2)
Postoperative ileus	1 (0.2)
Obstetric hysterectomy	1 (0.2)

Data expressed as *n* (%)

*FHR* fetal heart rate, *DBC* double-balloon catheter

### Bivariate analysis

Absence of previous vaginal delivery, history of intrapartum dystocia in previous pregnancy, and suspected fetal macrosomia (defined as estimated fetal weight >90th centile by third trimester ultrasound) increased two to three times the risk of a cesarean delivery (Table 4).

**Table 4:** Bivariate analysis (*n* = 386)

	Vaginal delivery	Cesarean section	OR (CI 95%)	*p*
Baseline characteristics				
Maternal age^a^	33 (6)	34.5 (7)	–	0.181
No previous vaginal delivery^b^	176 (81.9)	190 (93.6)	3.239 (1.673–6.268)	<0.001
Dystocia^c^ as indication for PCS^b^	74 (36.6)	105 (55.0)	2.145 (1.433–3.211)	<0.001
PCS without TOL^b^	74 (36.6)	57 (29.5)	NS	0.134
Suspected fetal macrosomia^b^	11 (5.1)	25 (12.3)	2.605 (1.246–5.443)	0.009
Gestational age (days)^a^	285.5 (8)	287 (1)	–	0.179
Maternal weight gain^a^	10 (7)	9.5 (7)	–	0.573
Initial Bishop score^a^	2 (2)	2.5 (1)	–	0.001
Preinduction outcomes				
Bishop score after DBC^a^	6 (4)	5.5 (3)	–	<0.001
Change in Bishop score^a^	4 (3)	3 (3)	–	<0.001
Absence of cervical changes^b^	14 (6.5)	30 (14.8)	2.490 (1.279–4.847)	0.006
Delivery outcomes				
Oxytocin induction^b^	152 (70.7)	179 (88.2)	3.091 (1.842–5.186)	<0.001
Induction period (min)^d^	133.43 ± 137.53	177.50 ± 156.78	–	<0.001
Active dilation period (min)^d^	226.35 ± 114.14	256.88 ± 131.33	–	<0.001
Second stage of labour (min)^d^	95.62 ± 56.49	128.44 ± 56.38	–	<0.001
Intrapartum maternal fever^b^	26 (12.1)	18 (8.9)	NS	0.283
Meconial amniotic fluid^b^	27 (12.6)	30 (14.8)	NS	0.509
Non-reassuring intrapartum FHR^b^	50 (23.3)	40 (19.7)	NS	0.377
Neonatal outcomes				
Birthweight^a^	3308.91 ± 474.10	3641.31 ± 296.30	–	<0.001
Apgar Test 5′ < 7^b^	2 (0.9)	2 (1.0)	NS	0.954
pH umbilical cord < 7.00^b^	2 (0.9)	3 (1.5)	NS	0.607
NICU admission^b^	2 (0.9)	3 (1.5)	NS	0.607
Maternal outcomes				
Anemia (<11 g/dl)^b^	28 (13.0)	121 (59.6)	9.855 (6.061–16.024)	<0.001
Uterine rupture^b^	3 (1.4)	2 (1.0)	NS	0.700

Bold highlight those *p* with stadistical significance

*FHR* fetal heart rate, *DBC* double-balloon catheter, *TOL* trial of labour, *NICU* neonatal intensive care unit, *NS* non-significant

^a^Data expressed as median (interquartile range)

^b^Data expressed as *n* (%)

^c^Dystocia included failed induction, failure to progress, or suspected intrapartum cephalopelvic disproportion

^d^Data expressed as mean ± SD

Bishop score before preinduction was higher in women who finally had a cesarean delivery, although that trend inverted when we analysed Bishop score after the removal of the mechanical device. In addition, those women who did not improved its cervical score had a 2.5-fold risk of repeated cesarean section.

As we expected, women with prolonged induction, active labour, and expulsion periods had higher rates of cesarean delivery. Higher birthweights and anemia rates were found in those women with a repeated cesarean section (Table 4).

### Multivariate model

This model was created to establish which antepartum variables were associated with a higher probability of having an intrapartum cesarean delivery because of dystocia. Although variables as Bishop score before and after preinduction had a statistical correlation with cesarean section rates in bivariate analysis, they were not relevant in multivariate design. Maternal age, gestational age, change in Bishop score, and need of oxytocin induction did not reached statistical significance in multivariate analysis and they were excluded from the final predictive model.

The best prediction model for intrapartum cesarean section included the following variables: dystocia in previous pregnancy (OR 1.744; *p* 0.026; CI 95% 1.066–2.846), absence of previous vaginal delivery (OR 2.590; *p* 0.036; CI 95% 1.066–6.290), suspected fetal macrosomia (OR 2.410; *p* 0.061; CI 95% 0.959–6.054), and duration of oxytocin induction period (OR 1.005; *p* 0.001; CI 95% 1.004–1.006) (Table 5). The area under the curve was 0.789 (*p* < 0.001). Hosmer and Lemeshow test was not significant and Nagelkerke *R* ^2^ was 0.339.

**Table 5:** Multivariate analysis for predicting intrapartum cesarean delivery (*n* = 386)

Variables	*p*	OR	CI 95%
Dystocia^a^ as indication for PCS (yes/no)	0.026	1.744	1.069–2.846
Absence of previous vaginal delivery (yes/no)	0.036	2.590	1.066–6.290
Suspected fetal macrosomia (yes/no)	0.061	2.410	0.959–6.054
Duration of oxytocin induction (min)	0.001	1.005	1.004–1.006

*PCS* previous cesarean section

^a^Dystocia included failed induction, failure to progress, or suspected intrapartum cephalopelvic disproportion

## Discussion

### Interpretation of main findings

#### Comparison with previous literature

We present our results about cervical ripening with DBC in women with PCS, being the largest study published to date. A few number of studies have shown their results using DBC in women with PCS (Table 6). Our study shows that the use of this device can be useful and safe in this clinical context. This could mean an important step for a change in the obstetric approach for women with a uterine scar.

**Table 6:** References about the use of DBC in women with a PCS

References	Year	*n*	Previous vaginal delivery (%)	Filling volume (ml)	Time of exposure (hours)	Initial Bishop score	Bishop after DBC	Change in Bishop score	Vaginal delivery rate (%)
Khotaba [13]	2001	34	2.7^c^	80	12	<4	–	6.2^b^	70.6
Miller [14]	2005	8	12.5	60	12	3.25^b^	5.87^b^	2.62^b^	25.0
Ferradas [15]	2013	32	–	20–80	12	1.16 ± 1.30^b^	3.22 ± 2.03^b^	2.06^b^	56.3
Ebeid [16]	2013	17	–	80	12	3^b^	6^b^	3^b^	52.9
Rossard [9]	2013	39	2.5^c^	80	24	3.5 ± 1.2^b^	5.4 ± 1.5^b^	1.84^b^	64.1
Boyon [11]	2014	50	64.0	80	12	3.3 ± 1.3^b^	5.4^b^	2.1 ± 1.8^b^	72.0
Cheuk [17]	2015	24	16.7	40–60	12	3 (3–4)^a^	7 (5.3–8)^a^	3 (2–4)^a^	75.0
De Bonrostro	2016	418	10.7	80	12–24	2 (2)^a^	5 (3)^a^	3 (2)^a^	51.4

*DBC* double-balloon catheter, *PCS* previous cesarean section

^a^Data expressed as median (interquartile range)

^b^Data expressed as mean ± SD

^c^Mean parity (percentage of patients with previous vaginal delivery not shown in the article)

High vaginal delivery rates (>70%) are described in several studies [11, 13, 17], probably related with a higher proportion of women with previous vaginal delivery and higher Bishop scores at the beginning of the preinduction process. Our results show a lower uterine rupture rate compared with studies using prostaglandins for induction of labour. Some authors have described uterine rupture rates with dinoprostone induction between 0 and 5.9% [6, 18]. Our results revealed a rate of uterine rupture similar to other studies about mechanical methods in cervical ripening [19, 20].

The diagnosis of intrapartum fever (axillary temperature ≥38 °C) was made in 10.5% of the women. This rate is reasonably low in spite of two major facts: a high proportion of women using epidural analgesia (87.25–90.03% during the recruitment period) and induction of labour by itself (higher number of vaginal examinations and longer periods of time until delivery). In any case, maternal fever rate has been reported to be developed up to 19.2% of women who have epidural anesthesia during labour [21]. Therefore, we did not find an increased risk of intrapartum fever in women using DBC for induction of labour.

Fetal blood sample testing was required in a high proportion of women included in this study. In our hospital, this technique for assessing intrapartum fetal well-being is needed in 10% of all deliveries. The increased rate observed in our study could be related to the fact that we are dealing with a high-risk pregnant women population: postterm pregnancies and labour induction are major risk factors for fetal non-reassuring patterns.

Although some authors have shown a change in tocogram pattern such as hyperstimulation preceding uterine rupture that may increase the rates of fetal heart rate abnormalities, uterine rupture was rare in our study, and it hardly could consistently rise the number of fetal blood tests [22].

In contrast, cesarean section for suspected fetal distress is very low regarding our results. Moreover, umbilical cord pH at birth and Apgar score show that the risk of fetal acidosis is not increased, since an appropriate intrapartum fetal well-being assessment is guaranteed.

All these data suggest that DBC seems to be an effective method for cervical ripening in women with a PCS with a probable reduction in the rate of uterine rupture compared with dinoprostone induction. There are several retrospective studies with Foley catheter in women with a PCS that show that is safe and effective for cervical ripening [19, 20]. However, Foley catheter is not a device designed for cervical ripening and its use regarding its data sheet is restricted to urological procedures.

#### Prediction models

Multivariate analysis showed that some clinical variables that can be easily obtained from medical history can help us predict the outcomes of the induction process, leading to a statistical model that will aid in taking clinical decisions during the TOL in women with a PCS.

In other studies, about pharmacological and mechanical methods of induction some maternal variables were associated to a higher rate of cesarean section, such as maternal Body Mass Index, maternal age, and Bishop score [23, 24]. However, although we found statistical association between Bishop score before and after preinduction and vaginal delivery rate in bivariate analysis, these variables were not relevant in multivariate model. In our study, we could not find differences in vaginal delivery rate regarding maternal age or maternal weight gain during pregnancy.

Several investigations have revealed that some ultrasonographic cervical parameters can have a potential for predicting the risk of cesarean section. Cheung found that transvaginal cervical length, maternal height, and posterior cervical angle could be predictable of a cesarean section in women with induction of labour, creating a multivariate model that reached an area under the curve of 0.79 [25]. In our study, although we did not assess ultrasonographic data, we could reach a similar predictability with our clinical model.

A recent review regarding different methods to predict vaginal delivery after preinduction of labour did not found enough evidence to support the use of transvaginal ultrasound over standard digital examination [26]. Bishop score seems to be a good predictor of vaginal delivery in preinduced labours. Our statistical model revealed that medical history can have a moderate predictive value for repeated cesarean section in women preinduced with DBC.

Some calculators have been created to assess the probability of a repeated cesarean section in women in TOL. It has been proven that the combination of several clinical variables can be helpful to determine the risk of having a repeated cesarean section in these cases [27]. Since there are no multivariate models for women with prior uterine scar undergoing cervical ripening with mechanical methods of induction, our investigation increases the options to assess the probability of having a vaginal delivery in new clinical situations.

### Strengths

Beyond the clinical relevance of our findings, this study have been designed carefully to avoid different bias. The high number of women included for statistical analysis and the selection criteria are the two mainstay of our investigation. Data collection was performed by one investigator to avoid heterogeneity in the information recorded.

All the women were attended in one hospital to reduce the variability in medical protocols regarding induction process associated with multicenter designs. Furthermore, selection criteria were established to reduce confounding factors, excluding clinical contexts that have a different obstetric approach during preinduction and labour progress (for example, multiple pregnancy, stillbirth or premature labour).

For bivariate and multivariate analysis, we decided to exclude those women with cesarean section in suspected fetal distress to isolate those factors associated with dystocia and minimize confounders that could interfere with the final result.

There are two main characteristics that make our study innovative: first, a comparatively large sample size on the use of double-balloon catheter for induction of labour in women with a previous uterine scar, an issue nearly unexplored in scientific literature. Moreover, we created a simple clinical prediction model for those women that initiated that mechanical preinduction process, being the first prediction tool for this mechanical device with a remarkable accuracy.

### Limitations

Unfortunately, sample size could be limited to assess accurately infrequent complications associated with TOL in women with a PCS (such as uterine rupture). Besides, we do not compare DBC with other preinduction methods what reduces the possibility of extrapolating differences that could exist between them. On the other hand, our results are concerning a group of women that were assisted in our hospital, and results of the process may be different in other centres with a change in clinical approach.

## Conclusions

Our data suggest that DBC seems to be a safe and effective method for induction of labour for women with a PCS with a low rate of uterine rupture. Some clinical variables such as parity and previous dystocia can estimate the risk of a repeated cesarean delivery with a moderate predictability. High-quality prospective and randomized trials comparing DBC with other pharmacological and mechanical methods are needed to confirm our data.

## Abbreviations

DBC: Double-balloon catheter
TOL: Trial of labour
PCS: Prior cesarean section
